# Author Correction: Sensing the DNA-mismatch tolerance of catalytically inactive Cas9 via barcoded DNA nanostructures in solid-state nanopores

**DOI:** 10.1038/s41551-023-01099-x

**Published:** 2023-09-04

**Authors:** Sarah E. Sandler, Nicole E. Weckman, Sarah Yorke, Akashaditya Das, Kaikai Chen, Richard Gutierrez, Ulrich F. Keyser

**Affiliations:** 1 Cavendish Laboratory, University of Cambridge, Cambridge, UK; 2 Department of Pathology, University of Cambridge, Cambridge, UK; 3 Oxford Nanopore Technologies, Oxford Science Park, Oxford, UK; 4 Present Address: Institute for Studies in Transdisciplinary Engineering Education & Practice, Department of Chemical Engineering & Applied Chemistry, University of Toronto, Toronto, Canada; 5 Present Address: Yusuf Hamied Department of Chemistry, Cambridge, UK; 6 Present Address: Department of Chemical Engineering, Imperial College London, London, UK

**Keywords:** Nanopores, Single-molecule biophysics, Biosensors, DNA nanotechnology, CRISPR-Cas systems

Correction to: *Nature Biomedical Engineering* 10.1038/s41551-023-01078-2. Published online 7 August 2023.

In the version of this article initially published, the surname of Richard Gutierrez appeared as “Guitterez”. The spelling has been corrected in the HTML and PDF versions of the article.

