# Supplementary material for: Sensing the DNA-mismatch tolerance of catalytically inactive Cas9 via barcoded DNA nanostructures in solid-state nanopores
Source: Nat Biomed Eng. 2023 Aug 7;8(3):325–34. doi: 10.1038/s41551-023-01078-2 (PMC10963265; doi:10.1038/s41551-023-01078-2)
Supplement: Supplementary file 1 — Supplementary figures, tables and references. [file 41551_2023_1078_MOESM1_ESM.pdf]

nature biomedical engineering

Article

https://doi.org/10.1038/s41551-023-01078-2

Sensing the DNA-mismatch tolerance of catalytically inactive Cas9 via barcoded DNA nanostructures in solid-state nanopores

In the format provided by the authors and unedited

Contents

Supplementary Table 1 l DNA Sequences '1' bits for DNA nanostructure.
Supplementary Table 2 l Sequences of DNA overhangs (related to Fig. 2).
Supplementary Table 3 l Sequences of DNA overhangs (related to Fig. 4).
Supplementary Table 4 l Sequences of DNA overhangs (related to Fig. 3).
Supplementary Table 5 l DNA dumbbell sequences (related to Fig. 3).

Supplementary Fig. 1 l Nanopore raw current trace.
Supplementary Fig. 2 l Event translocation highlighting asymmetry.
Supplementary Fig. 3 l Binding efficiency of the dCas9 probe (related to Fig. 3).
Supplementary Fig. 4 l DNA nanostructures in different salt conditions.
Supplementary Fig. 5 l Effect of time on the binding efficiency of dCas9 in different salt conditions.
Supplementary Fig. 6 l Folded vs. unfolded DNA translocations.

References

Supplementary Table 1 I Replacements to generate ‘1’ in barcodes.

The following replacements are made to create the “1” bits in the barcode portion of the nanostructure, as previously shown and used in Fig. 1 (ref. [1]).

First bit
Replace oligos 26,27,28,29,30,31 and 32

CTGAAAGCGTAAGAATACGTGGCACAGACAATATTTTTGAATGGCT
ACATCACTTGTCCTCTTTTGAGGAACAAGTTTTCTTGTCCTGAGTAGA
AGAACTCAAATCCTCTTTTGAGGAACAAGTTTTCTTGTCTATCGGCCT
TGCTGGTAATTCCTCTTTTGAGGAACAAGTTTTCTTGTATCCAGAACA
ATATTACCGCTCCTCTTTTGAGGAACAAGTTTTCTTGTCAGCCATTGC
AACAGGAAAATCCTCTTTTGAGGAACAAGTTTTCTTGTACGCTCATGG
AAATACCTACTCCTCTTTTGAGGAACAAGTTTTCTTGTATTTTGACGC
TCAATCGTCTTCCTCTTTTGAGGAACAAGTTTTCTTGTGAAATGGATT
ATTTACATTGTCCTCTTTTGAGGAACAAGTTTTCTTGTGCAGATTCAC
CAGTCACACGTCCTCTTTTGAGGAACAAGTTTTCTTGTACCAGTAATA
AAAGGGACATTCCTCTTTTGAGGAACAAGTTTTCTTGTTCTGGCCAAC
AGAGATAGAATCCTCTTTTGAGGAACAAGTTTTCTTGTCCCTTCTGAC

Second bit
Replace oligos 40,41,42,43,44,45 and 46

AATATAATCCTGATTGTTTGGATTATACTTCTGAATAATGGAAGGG
CACTAACAACTCCTCTTTTGAGGAACAAGTTTTCTTGTTAATAGATTA
GAGCCGTCAATCCTCTTTTGAGGAACAAGTTTTCTTGTTAGATAATAC
ATTTGAGGATTCCTCTTTTGAGGAACAAGTTTTCTTGTTTAGAAGTAT
TAGACTTTACTCCTCTTTTGAGGAACAAGTTTTCTTGTAAACAATTCG
ACAACTCGTATCCTCTTTTGAGGAACAAGTTTTCTTGTTTAAATCCTT
TGCCCGAACGTCCTCTTTTGAGGAACAAGTTTTCTTGTTTATTAATTT
TAAAAGTTTGTCCTCTTTTGAGGAACAAGTTTTCTTGTAGTAACATTA
TCATTTTGCGTCCTCTTTTGAGGAACAAGTTTTCTTGTGAACAAAGAA
ACCACCAGAATCCTCTTTTGAGGAACAAGTTTTCTTGTGGAGCGGAAT
TATCATCATATCCTCTTTTGAGGAACAAGTTTTCTTGTTTCCTGATTA
TCAGATGATGTCCTCTTTTGAGGAACAAGTTTTCTTGTGCAATTCATC

Third bit
Replace oligos 54,55,56,57,58,59 and 60

TGTAAATGCTGATGCAAATCCAATCGCAAGACAAAGAACGCGAGAA
TACCTTTTTTTCCTCTTTTGAGGAACAAGTTTTCTTGTAATGGAAACA
GTACATAAATTCCTCTTTTGAGGAACAAGTTTTCTTGTCAATATATGT
GAGTGAATAATCCTCTTTTGAGGAACAAGTTTTCTTGTCCTTGCTTCT
GTAAATCGTCTCCTCTTTTGAGGAACAAGTTTTCTTGTGCTATTAATT
AATTTTCCCTTCCTCTTTTGAGGAACAAGTTTTCTTGTTAGAATCCTT
GAAAACATAGTCCTCTTTTGAGGAACAAGTTTTCTTGTCGATAGCTTA
GATTAAGACGTCCTCTTTTGAGGAACAAGTTTTCTTGTCTGAGAAGAG
TCAATAGTGATCCTCTTTTGAGGAACAAGTTTTCTTGTATTTATCAAA
ATCATAGGTCTCCTCTTTTGAGGAACAAGTTTTCTTGTTGAGAGACTA
CCTTTTTAACTCCTCTTTTGAGGAACAAGTTTTCTTGTCTCCGGCTTA
GGTTGGGTTATCCTCTTTTGAGGAACAAGTTTTCTTGTTATAACTATA

Fourth bit
Replace oligos 68,69,70,71,72,73 and 74

TCATCGAGAACAAGCAAGCCGTTTTTATTTTCATCGTAGGAATCAT
AGAATATAAATCCTCTTTTGAGGAACAAGTTTTCTTGTGTACCGACAA
AAGGTAAAGTTCCTCTTTTGAGGAACAAGTTTTCTTGTAATTCTGTCC
AGACGACGACTCCTCTTTTGAGGAACAAGTTTTCTTGTAATAAACAAC
ATGTTCAGCTTCCTCTTTTGAGGAACAAGTTTTCTTGTAATGCAGAAC
GCGCCTGTTTTCCTCTTTTGAGGAACAAGTTTTCTTGTATCAACAATA
GATAAGTCCTTCCTCTTTTGAGGAACAAGTTTTCTTGTGAACAAGAAA
AATAATATCCTCCTCTTTTGAGGAACAAGTTTTCTTGTCATCCTAATT
TACGAGCATGTCCTCTTTTGAGGAACAAGTTTTCTTGTTAGAAACCAA
TCAATAATCGTCCTCTTTTGAGGAACAAGTTTTCTTGTGCTGTCTTTC
CTTATCATTCTCCTCTTTTGAGGAACAAGTTTTCTTGTCAAGAACGGG
TATTAAACCATCCTCTTTTGAGGAACAAGTTTTCTTGTAGTACCGCAC

Fifth bit

Replace oligos 82,83,84,85,86,87 and 88

AGATAGCCGAACAAAGTTACCAGAAGGAAACCGAGGAAACGCAATA
AAAAATGAAATCCTCTTTTGAGGAACAAGTTTTCTTGTATAGCAGCCT
TTACAGAGAGTCCTCTTTTGAGGAACAAGTTTTCTTGTAATAACATAA
AAACAGGGAATCCTCTTTTGAGGAACAAGTTTTCTTGTGCGCATTAGA
CGGGAGAATTTCCTCTTTTGAGGAACAAGTTTTCTTGTAACTGAACAC
CCTGAACAAATCCTCTTTTGAGGAACAAGTTTTCTTGTGTCAGAGGGT
AATTGAGCGCTCCTCTTTTGAGGAACAAGTTTTCTTGTTAATATCAGA
GAGATAACCCTCCTCTTTTGAGGAACAAGTTTTCTTGTACAAGAATTG
AGTTAAGCCCTCCTCTTTTGAGGAACAAGTTTTCTTGTAATAATAAGA
GCAAGAAACATCCTCTTTTGAGGAACAAGTTTTCTTGTATGAAATAGC
AATAGCTATCTCCTCTTTTGAGGAACAAGTTTTCTTGTTTACCGAAGC
CCTTTTTAAGTCCTCTTTTGAGGAACAAGTTTTCTTGTAAAAGTAAGC

Supplementary Table 2 l Sequences of overhangs for Fig. 2, the 50bp target sequence where the probe binds is in red.

Probe	Sequence
Green_142	GATGGTTTAATTTCAACTTTAATCATTGTGAATTACCTTTCGTATGGCAC CGGAACCGGTAAGGACGCGATCACCAGCGGCATCGAGGTC
Green_143	GACCTCGATGCCGCTGGTGATCGCGTCCTTACCGGTTCCGGTGCCATA CGTTTATGCGATTTTAAGAACTGGCTCATTATACCAGTCAG
Purple_142	GATGGTTTAATTTCAACTTTAATCATTGTGAATTACCTTTCGTATGGCAC CGGAACCGGTAAGGACGCGATCACCACCGGCATCGAGGTC
Purple_143	GACCTCGATGCCGGTGGTGATCGCGTCCTTACCGGTTCCGGTGCCATA CGTTTATGCGATTTTAAGAACTGGCTCATTATACCAGTCAGG
Control_142	GATGGTTTAATTTCAACTTTAATCATTGTGAATTACCTTTCTCTGATGAGT CGAGTCATTCGAGTGCTCTGAGTATCTTTTAAAGAGCAG
Control_143	CTGCTCTTTAAAAGATACTCAGAGCACTCGAATGACTCGACTCATCAGA GTTTATGCGATTTTAAGAACTGGCTCATTATACCAGTCAGG

Supplementary Table 3 l Sequences of overhangs for Fig. 4.

Probe	Sequence
WT_142	GATGGTTTAATTTCAACTTTAATCATTGTGAATTACCTTTTTAAATAGAGCAAATCCCCTTATT GGGGGTAAGACATGAAGATGCCAGAA
WT_143	TTCTGGCATCTTCATGTCTTACCCCCAATAAGGGGATTTGCTCTATTTAATTTATGCGATTTT AAGAACTGGCTCATTATACCAGTCAGG
Mut1_142	GATGGTTTAATTTCAACTTTAATCATTGTGAATTACCTTTTTAAATAGAGCAAATCCCCGTATT GGGGGTAAGACATGAAGATGCCAGAA
Mut1_143	TTCTGGCATCTTCATGTCTTACCCCCAATACGGGGATTTGCTCTATTTAATTTATGCGATTTT AAGAACTGGCTCATTATACCAGTCAGG
Mut2_142	GATGGTTTAATTTCAACTTTAATCATTGTGAATTACCTTTTTAAATAGAGCAAATCCCCTGATT GGGGGTAAGACATGAAGATGCCAGAA
Mut2_143	TTCTGGCATCTTCATGTCTTACCCCCAATCAGGGGATTTGCTCTATTTAATTTATGCGATTTT AAGAACTGGCTCATTATACCAGTCAGG
Mut3_142	GATGGTTTAATTTCAACTTTAATCATTGTGAATTACCTTTTTAAATAGAGCAAATCCCCTTCTT GGGGGTAAGACATGAAGATGCCAGAA
Mut3_143	TTCTGGCATCTTCATGTCTTACCCCCAAGAAGGGGATTTGCTCTATTTAATTTATGCGATTTT AAGAACTGGCTCATTATACCAGTCAGG
Mut4_142	GATGGTTTAATTTCAACTTTAATCATTGTGAATTACCTTTTTAAATAGAGCAAATCCCCTTAGT GGGGGTAAGACATGAAGATGCCAGAA
Mut4_143	TTCTGGCATCTTCATGTCTTACCCCCACTAAGGGGATTTGCTCTATTTAATTTATGCGATTTT AAGAACTGGCTCATTATACCAGTCAGG

Supplementary Table 4 l Sequences of Overhangs for Nanostructure in Fig. 3.

Name	Sequence
WT_44	ACAAAGAAACCACCAGAAGGAGCGGAATTATCATCATATTCGTATGGCACCGG ATTTGGTAAGGACGCGATCACCAGCGGCATCGAGGTC
WT_45	GACCTCGATGCCGCTGGTGATCGCGTCCTTACCAAATCCGGTGCCATACGTTT TCCTGATTATCAGATGATGGCAATTCATCAATATAAT
WT_81	TCCCAATCCAAATAAGAAACGATTTTTTGTTTAACGTCTTCGTATGGCACCGGA TTTGGTAAGGACGCGATCACCAGCGGCATCGAGGTC
WT_82	GACCTCGATGCCGCTGGTGATCGCGTCCTTACCAAATCCGGTGCCATACGTTA AAAATGAAAATAGCAGCCTTTACAGAGAGAATAACAT
WT_118	TCGTCACCAGTACAAACTACAACGCCTGTAGCATTCCATTCGTATGGCACCGG ATTTGGTAAGGACGCGATCACCAGCGGCATCGAGGTC
WT_119	GACCTCGATGCCGCTGGTGATCGCGTCCTTACCAAATCCGGTGCCATACGTT CAGACAGCCCTCATAGTTAGCGTAACGATCTAAAGTTT
PAMC_A_44	ACAAAGAAACCACCAGAAGGAGCGGAATTATCATCATATTCGTATGGCACCGG ATTTGGTAAGGACGCGATCACCAGCTGCATCGAGGTC
PAMC_A_45	GACCTCGATGCAGCTGGTGATCGCGTCCTTACCAAATCCGGTGCCATACGTTT TCCTGATTATCAGATGATGGCAATTCATCAATATAAT
PAMC_T_81	TCCCAATCCAAATAAGAAACGATTTTTTGTTTAACGTCTTCGTATGGCACCGGA TTTGGTAAGGACGCGATCACCAGCAGCATCGAGGTC
PAMC_T_82	GACCTCGATGCTGCTGGTGATCGCGTCCTTACCAAATCCGGTGCCATACGTTA AAAATGAAAATAGCAGCCTTTACAGAGAGAATAACAT
PAMC_G_118	TCGTCACCAGTACAAACTACAACGCCTGTAGCATTCCATTCGTATGGCACCGG ATTTGGTAAGGACGCGATCACCAGCCGCATCGAGGTC
PAMC_G_119	GACCTCGATGCGGCTGGTGATCGCGTCCTTACCAAATCCGGTGCCATACGTT CAGACAGCCCTCATAGTTAGCGTAACGATCTAAAGTTT
1C_A_44	ACAAAGAAACCACCAGAAGGAGCGGAATTATCATCATATTCGTATGGCACCGG ATTTGGTAAGGACGCGATCACCATCGGCATCGAGGTC
1C_A_45	GACCTCGATGCCGATGGTGATCGCGTCCTTACCAAATCCGGTGCCATACGTTT TCCTGATTATCAGATGATGGCAATTCATCAATATAAT
1C_T_81	TCCCAATCCAAATAAGAAACGATTTTTTGTTTAACGTCTTCGTATGGCACCGGA TTTGGTAAGGACGCGATCACCAACGGCATCGAGGTC
1C_T_82	GACCTCGATGCCGTTGGTGATCGCGTCCTTACCAAATCCGGTGCCATACGTTA AAAATGAAAATAGCAGCCTTTACAGAGAGAATAACAT
1C_G_118	TCGTCACCAGTACAAACTACAACGCCTGTAGCATTCCATTCGTATGGCACCGG ATTTGGTAAGGACGCGATCACCACCGGCATCGAGGTC
1C_G_119	GACCTCGATGCCGGTGGTGATCGCGTCCTTACCAAATCCGGTGCCATACGTT CAGACAGCCCTCATAGTTAGCGTAACGATCTAAAGTTT
2T_A_44	ACAAAGAAACCACCAGAAGGAGCGGAATTATCATCATATTCGTATGGCACCGG ATTTGGTAAGGACGCGATCACCTGCGGCATCGAGGTC
2T_A_45	GACCTCGATGCCGCAGGTGATCGCGTCCTTACCAAATCCGGTGCCATACGTTT TCCTGATTATCAGATGATGGCAATTCATCAATATAAT
2T_G_81	TCCCAATCCAAATAAGAAACGATTTTTTGTTTAACGTCTTCGTATGGCACCGGA TTTGGTAAGGACGCGATCACCCGCGGCATCGAGGTC
2T_G_82	GACCTCGATGCCGCGGGTGATCGCGTCCTTACCAAATCCGGTGCCATACGTT AAAAATGAAAATAGCAGCCTTTACAGAGAGAATAACAT
2T_C_118	TCGTCACCAGTACAAACTACAACGCCTGTAGCATTCCATTCGTATGGCACCGG ATTTGGTAAGGACGCGATCACCGGCGGCATCGAGGTC
2T_C_119	GACCTCGATGCCGCCGGTGATCGCGTCCTTACCAAATCCGGTGCCATACGTT CAGACAGCCCTCATAGTTAGCGTAACGATCTAAAGTTT
3G_A_44	ACAAAGAAACCACCAGAAGGAGCGGAATTATCATCATATTCGTATGGCACCGG ATTTGGTAAGGACGCGATCACTAGCGGCATCGAGGTC
3G_A_45	GACCTCGATGCCGCTAGTGATCGCGTCCTTACCAAATCCGGTGCCATACGTTT TCCTGATTATCAGATGATGGCAATTCATCAATATAAT
3G_T_81	TCCCAATCCAAATAAGAAACGATTTTTTGTTTAACGTCTTCGTATGGCACCGGA TTTGGTAAGGACGCGATCACAAGCGGCATCGAGGTC
3G_T_82	GACCTCGATGCCGCTTGTGATCGCGTCCTTACCAAATCCGGTGCCATACGTTA AAAATGAAAATAGCAGCCTTTACAGAGAGAATAACAT

3G_C_118	TCGTCACCAGTACAAACTACAACGCCTGTAGCATTCCATTCGTATGGCACCGGATTTGGTAAGGACGCGATCACGAGCGGCATCGAGGTC
3G_C_119	GACCTCGATGCCGCTCGTGATCGCGTCCTTACCAAATCCGGTGCCATACGTTCAGACAGCCCTCATAGTTAGCGTAACGATCTAAAGTTT
4G_A_44	ACAAAGAAACCACCAGAAGGAGCGGAATTATCATCATATTCGTATGGCACCGGATTTGGTAAGGACGCGATCATCAGCGGCATCGAGGTC
4G_A_45	GACCTCGATGCCGCTGATGATCGCGTCCTTACCAAATCCGGTGCCATACGTTTTCCTGATTATCAGATGATGGCAATTCATCAATATAAT
4G_T_81	TCCCAATCCAAATAAGAAACGATTTTTTGTTTAACGTCTTCGTATGGCACCGGATTGGTAAGGACGCGATCAACAGCGGCATCGAGGTC
4G_T_82	GACCTCGATGCCGCTGTTGATCGCGTCCTTACCAAATCCGGTGCCATACGTTAAAAATGAAAATAGCAGCCTTTACAGAGAGAATAACAT
4G_C_118	TCGTCACCAGTACAAACTACAACGCCTGTAGCATTCCATTCGTATGGCACCGGATTTGGTAAGGACGCGATCAGCAGCGGCATCGAGGTC
4G_C_119	GACCTCGATGCCGCTGCTGATCGCGTCCTTACCAAATCCGGTGCCATACGTTCAGACAGCCCTCATAGTTAGCGTAACGATCTAAAGTTT
5T_A_44	ACAAAGAAACCACCAGAAGGAGCGGAATTATCATCATATTCGTATGGCACCGGATTTGGTAAGGACGCGATCTCCAGCGGCATCGAGGTC
5T_A_45	GACCTCGATGCCGCTGGAGATCGCGTCCTTACCAAATCCGGTGCCATACGTTTTCCTGATTATCAGATGATGGCAATTCATCAATATAAT
5T_G_81	TCCCAATCCAAATAAGAAACGATTTTTTGTTTAACGTCTTCGTATGGCACCGGATTTGGTAAGGACGCGATCCCCAGCGGCATCGAGGTC
5T_G_82	GACCTCGATGCCGCTGGGGATCGCGTCCTTACCAAATCCGGTGCCATACGTTAAAAATGAAAATAGCAGCCTTTACAGAGAGAATAACAT
5T_C_118	TCGTCACCAGTACAAACTACAACGCCTGTAGCATTCCATTCGTATGGCACCGGATTTGGTAAGGACGCGATCGCCAGCGGCATCGAGGTC
5T_C_119	GACCTCGATGCCGCTGGCGATCGCGTCCTTACCAAATCCGGTGCCATACGTTCAGACAGCCCTCATAGTTAGCGTAACGATCTAAAGTTT
6G_A_44	ACAAAGAAACCACCAGAAGGAGCGGAATTATCATCATATTCGTATGGCACCGGATTTGGTAAGGACGCGATTACCAGCGGCATCGAGGTC
6G_A_45	GACCTCGATGCCGCTGGTAATCGCGTCCTTACCAAATCCGGTGCCATACGTTTTCCTGATTATCAGATGATGGCAATTCATCAATATAAT
6G_T_81	TCCCAATCCAAATAAGAAACGATTTTTTGTTTAACGTCTTCGTATGGCACCGGATTTGGTAAGGACGCGATAACCAGCGGCATCGAGGTC
6G_T_82	GACCTCGATGCCGCTGGTTATCGCGTCCTTACCAAATCCGGTGCCATACGTTAAAAATGAAAATAGCAGCCTTTACAGAGAGAATAACAT
6G_C_118	TCGTCACCAGTACAAACTACAACGCCTGTAGCATTCCATTCGTATGGCACCGGATTTGGTAAGGACGCGATGACCAGCGGCATCGAGGTC
6G_C_119	GACCTCGATGCCGCTGGTCATCGCGTCCTTACCAAATCCGGTGCCATACGTTCAGACAGCCCTCATAGTTAGCGTAACGATCTAAAGTTT
7A_T_44	ACAAAGAAACCACCAGAAGGAGCGGAATTATCATCATATTCGTATGGCACCGGATTTGGTAAGGACGCGAACACCAGCGGCATCGAGGTC
7A_T_45	GACCTCGATGCCGCTGGTGTTCGCGTCCTTACCAAATCCGGTGCCATACGTTTTCCTGATTATCAGATGATGGCAATTCATCAATATAAT
7A_G_81	TCCCAATCCAAATAAGAAACGATTTTTTGTTTAACGTCTTCGTATGGCACCGGATTTGGTAAGGACGCGACCACCAGCGGCATCGAGGTC
7A_G_82	GACCTCGATGCCGCTGGTGGTCGCGTCCTTACCAAATCCGGTGCCATACGTTAAAAATGAAAATAGCAGCCTTTACAGAGAGAATAACAT
7A_C_118	TCGTCACCAGTACAAACTACAACGCCTGTAGCATTCCATTCGTATGGCACCGGATTTGGTAAGGACGCGAGCACCAGCGGCATCGAGGTC
7A_C_119	GACCTCGATGCCGCTGGTGCTCGCGTCCTTACCAAATCCGGTGCCATACGTTCAGACAGCCCTCATAGTTAGCGTAACGATCTAAAGTTT
8T_A_44	ACAAAGAAACCACCAGAAGGAGCGGAATTATCATCATATTCGTATGGCACCGGATTTGGTAAGGACGCGTTCACCAGCGGCATCGAGGTC
8T_A_45	GACCTCGATGCCGCTGGTGAACGCGTCCTTACCAAATCCGGTGCCATACGTTTTCCTGATTATCAGATGATGGCAATTCATCAATATAAT
8T_G_81	TCCCAATCCAAATAAGAAACGATTTTTTGTTTAACGTCTTCGTATGGCACCGGATTTGGTAAGGACGCGCTCACCAGCGGCATCGAGGTC

8T_G_82	GACCTCGATGCCGCTGGTGAGCGCGTCCTTACCAAATCCGGTGCCATACGTT AAAAATGAAAATAGCAGCCTTTACAGAGAGAATAACAT
8T_C_118	TCGTCACCAGTACAAACTACAACGCCTGTAGCATTCCATTCGTATGGCACCGG ATTTGGTAAGGACGCGGTCACCAGCGGCATCGAGGTC
8T_C_119	GACCTCGATGCCGCTGGTGACCGCGTCCTTACCAAATCCGGTGCCATACGTT CAGACAGCCCTCATAGTTAGCGTAACGATCTAAAGTTT
10G_A_44	ACAAAGAAACCACCAGAAGGAGCGGAATTATCATCATATTCGTATGGCACCGG ATTTGGTAAGGACGTGATCACCAGCGGCATCGAGGTC
10G_A_45	GACCTCGATGCCGCTGGTGATCACGTCCTTACCAAATCCGGTGCCATACGTTT TCCTGATTATCAGATGATGGCAATTCATCAATATAAT
10G_T_81	TCCCAATCCAAATAAGAAACGATTTTTTGTTTAACGTCTTCGTATGGCACCGGA TTTGGTAAGGACGAGATCACCAGCGGCATCGAGGTC
10G_T_82	GACCTCGATGCCGCTGGTGATCTCGTCCTTACCAAATCCGGTGCCATACGTTA AAAATGAAAATAGCAGCCTTTACAGAGAGAATAACAT
10G_C_118	TCGTCACCAGTACAAACTACAACGCCTGTAGCATTCCATTCGTATGGCACCGG ATTTGGTAAGGACGGGATCACCAGCGGCATCGAGGTC
10G_C_119	GACCTCGATGCCGCTGGTGATCCCGTCCTTACCAAATCCGGTGCCATACGTTC AGACAGCCCTCATAGTTAGCGTAACGATCTAAAGTTT
12G_A_44	ACAAAGAAACCACCAGAAGGAGCGGAATTATCATCATATTCGTATGGCACCGG ATTTGGTAAGGATGCGATCACCAGCGGCATCGAGGTC
12G_A_45	GACCTCGATGCCGCTGGTGATCGCATCCTTACCAAATCCGGTGCCATACGTTT TCCTGATTATCAGATGATGGCAATTCATCAATATAAT
12G_T_81	TCCCAATCCAAATAAGAAACGATTTTTTGTTTAACGTCTTCGTATGGCACCGGA TTTGGTAAGGAAGCGATCACCAGCGGCATCGAGGTC
12G_T_82	GACCTCGATGCCGCTGGTGATCGCTTCCTTACCAAATCCGGTGCCATACGTTA AAAATGAAAATAGCAGCCTTTACAGAGAGAATAACAT
12G_C_118	TCGTCACCAGTACAAACTACAACGCCTGTAGCATTCCATTCGTATGGCACCGG ATTTGGTAAGGAGGCGATCACCAGCGGCATCGAGGTC
12G_C_119	GACCTCGATGCCGCTGGTGATCGCCTCCTTACCAAATCCGGTGCCATACGTTC AGACAGCCCTCATAGTTAGCGTAACGATCTAAAGTTT
14C_A_44	ACAAAGAAACCACCAGAAGGAGCGGAATTATCATCATATTCGTATGGCACCGG ATTTGGTAAGTACGCGATCACCAGCGGCATCGAGGTC
14C_A_45	GACCTCGATGCCGCTGGTGATCGCGTACTTACCAAATCCGGTGCCATACGTTT TCCTGATTATCAGATGATGGCAATTCATCAATATAAT
14C_T_81	TCCCAATCCAAATAAGAAACGATTTTTTGTTTAACGTCTTCGTATGGCACCGGA TTTGGTAAGAACGCGATCACCAGCGGCATCGAGGTC
14C_T_82	GACCTCGATGCCGCTGGTGATCGCGTTCTTACCAAATCCGGTGCCATACGTTA AAAATGAAAATAGCAGCCTTTACAGAGAGAATAACAT
14C_G_118	TCGTCACCAGTACAAACTACAACGCCTGTAGCATTCCATTCGTATGGCACCGG ATTTGGTAAGCACGCGATCACCAGCGGCATCGAGGTC
14C_G_119	GACCTCGATGCCGCTGGTGATCGCGTGCTTACCAAATCCGGTGCCATACGTT CAGACAGCCCTCATAGTTAGCGTAACGATCTAAAGTTT
16T_A_44	ACAAAGAAACCACCAGAAGGAGCGGAATTATCATCATATTCGTATGGCACCGG ATTTGGTATGGACGCGATCACCAGCGGCATCGAGGTC
16T_A_45	GACCTCGATGCCGCTGGTGATCGCGTCCATACCAAATCCGGTGCCATACGTTT TCCTGATTATCAGATGATGGCAATTCATCAATATAAT
16T_G_81	TCCCAATCCAAATAAGAAACGATTTTTTGTTTAACGTCTTCGTATGGCACCGGA TTTGGTACGGACGCGATCACCAGCGGCATCGAGGTC
16T_G_82	GACCTCGATGCCGCTGGTGATCGCGTCCGTACCAAATCCGGTGCCATACGTT AAAAATGAAAATAGCAGCCTTTACAGAGAGAATAACAT
16T_C_118	TCGTCACCAGTACAAACTACAACGCCTGTAGCATTCCATTCGTATGGCACCGG ATTTGGTAGGGACGCGATCACCAGCGGCATCGAGGTC
16T_C_119	GACCTCGATGCCGCTGGTGATCGCGTCCCTACCAAATCCGGTGCCATACGTT CAGACAGCCCTCATAGTTAGCGTAACGATCTAAAGTTT

Supplementary Table 5 | Dumbbell Sequences for the nanostructure in Fig. 3.

Name	Sequence
Bit1_23_28	AACGGTACGCTCCTCTTTTGAGGAACAAGTTTTCTTGTCAGAATCCTG
Bit1_23_28	AGAAGTGTTTTCCTCTTTTGAGGAACAAGTTTTCTTGTTTATAATCAG
Bit1_23_28	TGAGGCCACCTCCTCTTTTGAGGAACAAGTTTTCTTGTGAGTAAAAGA
Bit1_23_28	GTCTGTCCATTCCTCTTTTGAGGAACAAGTTTTCTTGTCACGCAAATT
Bit1_23_28	AACCGTTGTATCCTCTTTTGAGGAACAAGTTTTCTTGTGCAATACTTC
Bit1_23_28	TTTGATTAGTTCCTCTTTTGAGGAACAAGTTTTCTTGTAATAACATCA
Bit1_23_28	CTTGCCTGAGTCCTCTTTTGAGGAACAAGTTTTCTTGTTAGAAGAACT
Bit1_23_28	CAAACTATCGTCCTCTTTTGAGGAACAAGTTTTCTTGTGCCTTGCTGG
Bit1_23_28	TAATATCCAGTCCTCTTTTGAGGAACAAGTTTTCTTGTAACAATATTA
Bit1_23_28	CCGCCAGCCATCCTCTTTTGAGGAACAAGTTTTCTTGTTTGCAACAGG
Bit1_23_28	AAAAACGCTCTCCTCTTTTGAGGAACAAGTTTTCTTGTATGGAAATACCTACATTT
Bit2_60_65	CTGATGCAAATCCTCTTTTGAGGAACAAGTTTTCTTGTTCCAATCGCA
Bit2_60_65	AGACAAAGAATCCTCTTTTGAGGAACAAGTTTTCTTGTCGCGAGAAAA
Bit2_60_65	CTTTTTCAAATCCTCTTTTGAGGAACAAGTTTTCTTGTTATATTTTAG
Bit2_60_65	TTAATTTCATTCCTCTTTTGAGGAACAAGTTTTCTTGTCTTCTGACCT
Bit2_60_65	AAATTTAATGTCCTCTTTTGAGGAACAAGTTTTCTTGTGTTTGAAATA
Bit2_60_65	CCGACCGTGTTCCTCTTTTGAGGAACAAGTTTTCTTGTGATAAATAAG
Bit2_60_65	GCGTTAAATATCCTCTTTTGAGGAACAAGTTTTCTTGTAGAATAAACA
Bit2_60_65	CCGGAATCATTCCTCTTTTGAGGAACAAGTTTTCTTGTAATTACTAGA
Bit2_60_65	AAAAGCCTGTTCCTCTTTTGAGGAACAAGTTTTCTTGTTTAGTATCAT
Bit2_60_65	ATGCGTTATATCCTCTTTTGAGGAACAAGTTTTCTTGTCAAATTCTTA
Bit2_60_65	CCAGTATAAATCCTCTTTTGAGGAACAAGTTTTCTTGTGCCAACGCTCAACAGTAG
Bit3_97_102	GTAGCACCATTCCTCTTTTGAGGAACAAGTTTTCTTGTTACCATTAGC
Bit3_97_102	AAGGCCGGAATCCTCTTTTGAGGAACAAGTTTTCTTGTACGTCACCAA
Bit3_97_102	TGAAACCATCTCCTCTTTTGAGGAACAAGTTTTCTTGTGATAGCAGCA
Bit3_97_102	CCGTAATCAGTCCTCTTTTGAGGAACAAGTTTTCTTGTTAGCGACAGA
Bit3_97_102	ATCAAGTTTGTCCTCTTTTGAGGAACAAGTTTTCTTGTCCTTTAGCGT
Bit3_97_102	CAGACTGTAGTCCTCTTTTGAGGAACAAGTTTTCTTGTCGCGTTTTCA
Bit3_97_102	TCGGCATTTTTCCTCTTTTGAGGAACAAGTTTTCTTGTCGGTCATAGC
Bit3_97_102	CCCCTTATTATCCTCTTTTGAGGAACAAGTTTTCTTGTGCGTTTGCCA
Bit3_97_102	TCTTTTCATATCCTCTTTTGAGGAACAAGTTTTCTTGTATCAAAATCA
Bit3_97_102	CCGGAACCAGTCCTCTTTTGAGGAACAAGTTTTCTTGTAGCCACCACC
Bit3_97_102	GGAACCGCCTTCCTCTTTTGAGGAACAAGTTTTCTTGTCCCTCAGAGCCGCCACCC
Bit4_134_139	CAAAGTACAATCCTCTTTTGAGGAACAAGTTTTCTTGTCGGAGATTTG
Bit4_134_139	TATCATCGCCTCCTCTTTTGAGGAACAAGTTTTCTTGTTGATAAATTG
Bit4_134_139	TGTCGAAATCTCCTCTTTTGAGGAACAAGTTTTCTTGTCGCGACCTGC
Bit4_134_139	TCCATGTTACTCCTCTTTTGAGGAACAAGTTTTCTTGTTTAGCCGGAA
Bit4_134_139	CGAGGCGCAGTCCTCTTTTGAGGAACAAGTTTTCTTGTACGGTCAATC
Bit4_134_139	ATAAGGGAACTCCTCTTTTGAGGAACAAGTTTTCTTGTCGAACTGACC
Bit4_134_139	AACTTTGAAATCCTCTTTTGAGGAACAAGTTTTCTTGTGAGGACAGAT
Bit4_134_139	GAACGGTGTATCCTCTTTTGAGGAACAAGTTTTCTTGTCAGACCAGGC
Bit4_134_139	GCATAGGCTGTCCTCTTTTGAGGAACAAGTTTTCTTGTGCTGACCTTC
Bit4_134_139	ATCAAGAGTATCCTCTTTTGAGGAACAAGTTTTCTTGTATCTTGACAA

Bit4_134_139	GAACCGGATATCCTCTTTTGAGGAACAAGTTTTCTTGTTTCATTACCCAAATCAAC
Bit5_148_153	CACTATCATATCCTCTTTTGAGGAACAAGTTTTCTTGTACCCTCGTTT
Bit5_148_140	ACCAGACGACTCCTCTTTTGAGGAACAAGTTTTCTTGTGATAAAAACC
Bit5_148_141	AAAATAGCGATCCTCTTTTGAGGAACAAGTTTTCTTGTGAGGCTTTTG
Bit5_148_142	CAAAAGAAGTTCCTCTTTTGAGGAACAAGTTTTCTTGTTTTGCCAGAG
Bit5_148_143	GGGGTAATAGTCCTCTTTTGAGGAACAAGTTTTCTTGTTAAAATGTTT
Bit5_148_144	AGACTGGATATCCTCTTTTGAGGAACAAGTTTTCTTGTGCGTCCAATA
Bit5_148_145	CTGCGGAATCTCCTCTTTTGAGGAACAAGTTTTCTTGTGTCATAAATA
Bit5_148_146	TTCATTGAATTCCTCTTTTGAGGAACAAGTTTTCTTGTCCCCCTCAAA
Bit5_148_147	TGCTTTAAACTCCTCTTTTGAGGAACAAGTTTTCTTGTAGTTCAGAAA
Bit5_148_148	ACGAGAATGATCCTCTTTTGAGGAACAAGTTTTCTTGTCCATAAATCA
Bit5_148_149	AAAATCAGGTTCCTCTTTTGAGGAACAAGTTTTCTTGTCTTTACCCTGACTATTAT
Bit6_162_167	TTGACCATTATCCTCTTTTGAGGAACAAGTTTTCTTGTGATACATTTC
Bit6_162_167	GCAAATGGTCTCCTCTTTTGAGGAACAAGTTTTCTTGTAATAACCTGT
Bit6_162_167	TTAGCTATATTCCTCTTTTGAGGAACAAGTTTTCTTGTTTTCATTTGG
Bit6_162_167	GGCGCGAGCTTCCTCTTTTGAGGAACAAGTTTTCTTGTGAAAAGGTGG
Bit6_162_167	CATCAATTCTTCCTCTTTTGAGGAACAAGTTTTCTTGTACTAATAGTA
Bit6_162_167	GTAGCATTAATCCTCTTTTGAGGAACAAGTTTTCTTGTCATCCAATAA
Bit6_162_167	ATCATACAGGTCCTCTTTTGAGGAACAAGTTTTCTTGTCAAGGCAAAG
Bit6_162_167	AATTAGCAAATCCTCTTTTGAGGAACAAGTTTTCTTGTATTAAGCAAT
Bit6_162_167	AAAGCCTCAGTCCTCTTTTGAGGAACAAGTTTTCTTGTAGCATAAAGC
Bit6_162_167	TAAATCGGTTTCCTCTTTTGAGGAACAAGTTTTCTTGTGTACCAAAAA
Bit6_162_167	CATTATGACCTCCTCTTTTGAGGAACAAGTTTTCTTGTCTGTAATACTTTTGCGGG

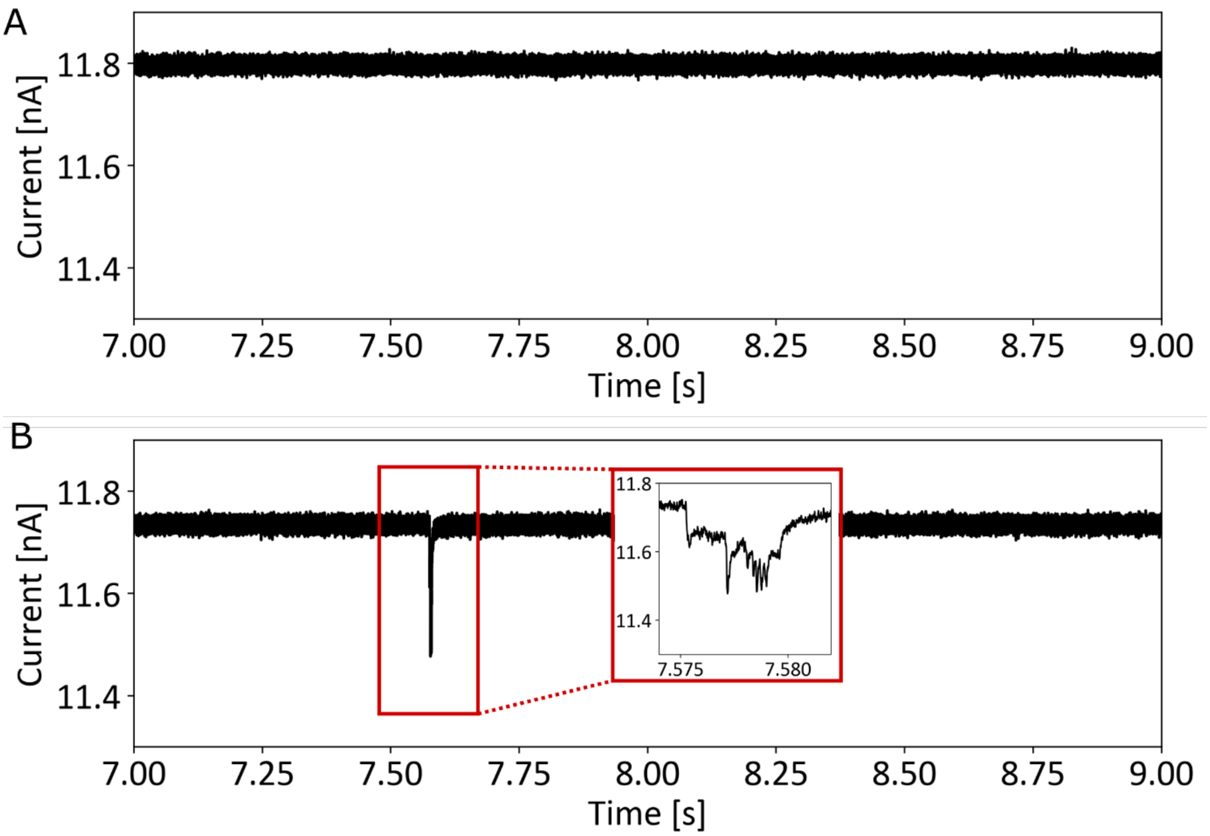

Supplementary Fig. 1 | Raw current trace in Nanopore in 4M LiCl for data show in Supplementary Fig. 5. (A) No DNA/protein is being detected in the pore (B) Current trace where DNA nanostructure with 11111 barcode and dCas9 bound is translocating.

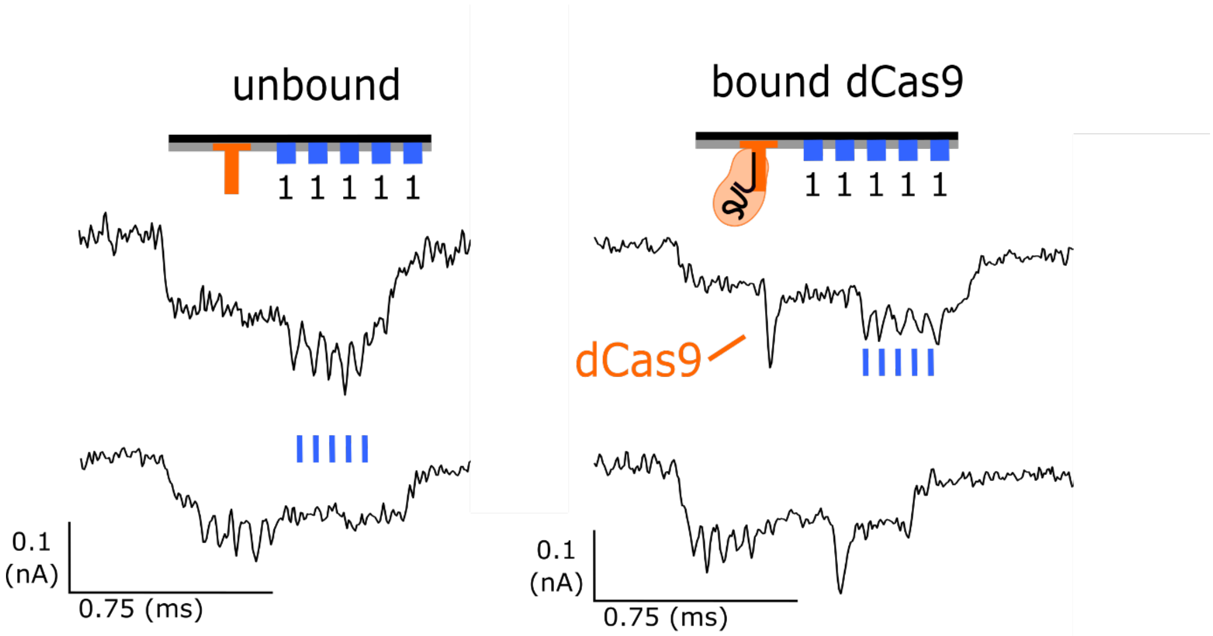

Supplementary Fig. 2 I Example events shown for the DNA nanostructure with dCas9 Probe 2 from Fig. 4 with a 11111 barcode without dCas9 (left) and with dCas9 (right).
The dCas9 binding to the dsDNA overhang creates an additional deeper spike in the second half of the event than in events with no dCas9. The events can translocate in either direction, thus design must asymmetric. Events were measured in 2M LiCl.

The second nanostructure, depicted in Fig. 3, multiplexes the binding to three possible binding sites on the same nanostructure. This structure was used to test the effect of the off-target binding at different positions and bases on the same structure for the experiments in Fig. 3. The design is asymmetric so that the positions with dCas9 bound during translocations can be distinguished when the DNA enters the pore from either direction as seen in the figure. The events were measured in 4M LiCl.

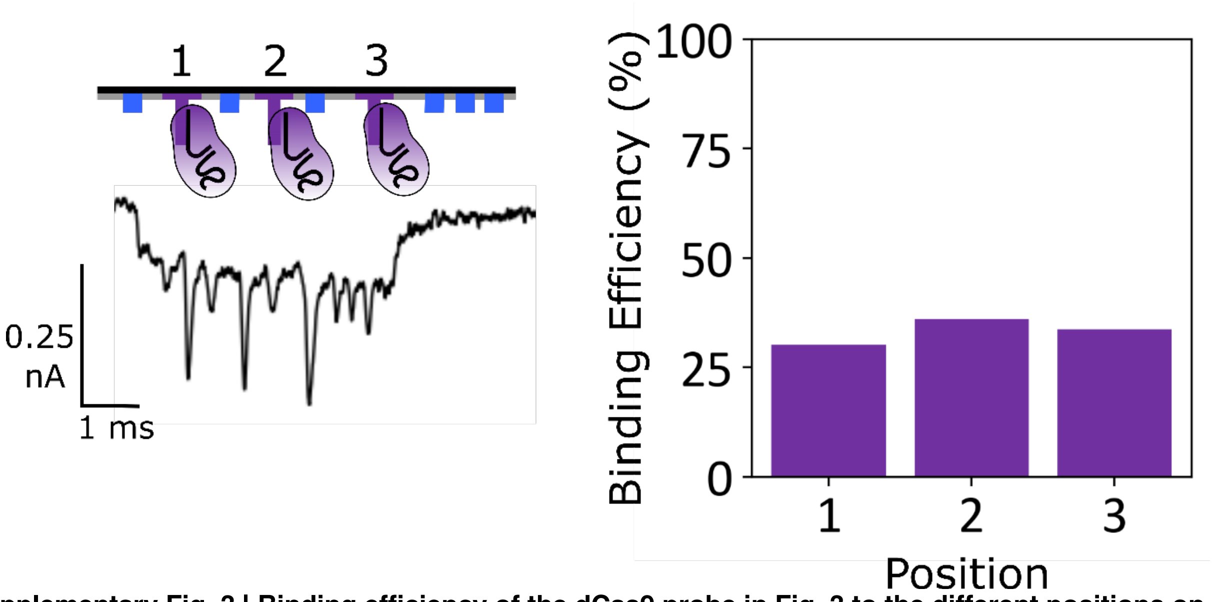

Supplementary Fig. 3 l Binding efficiency of the dCas9 probe in Fig. 3 to the different positions on the DNA nanostructures in Fig. 3. The standard deviation between the binding efficiency of the dCas9 probe to the different positions is 2.4%, the observed binding efficiency in each position is graphed in Supplementary Fig. 4. The average between the three positions, 33.7% acts as the normalization factor for the measurements for specificity.

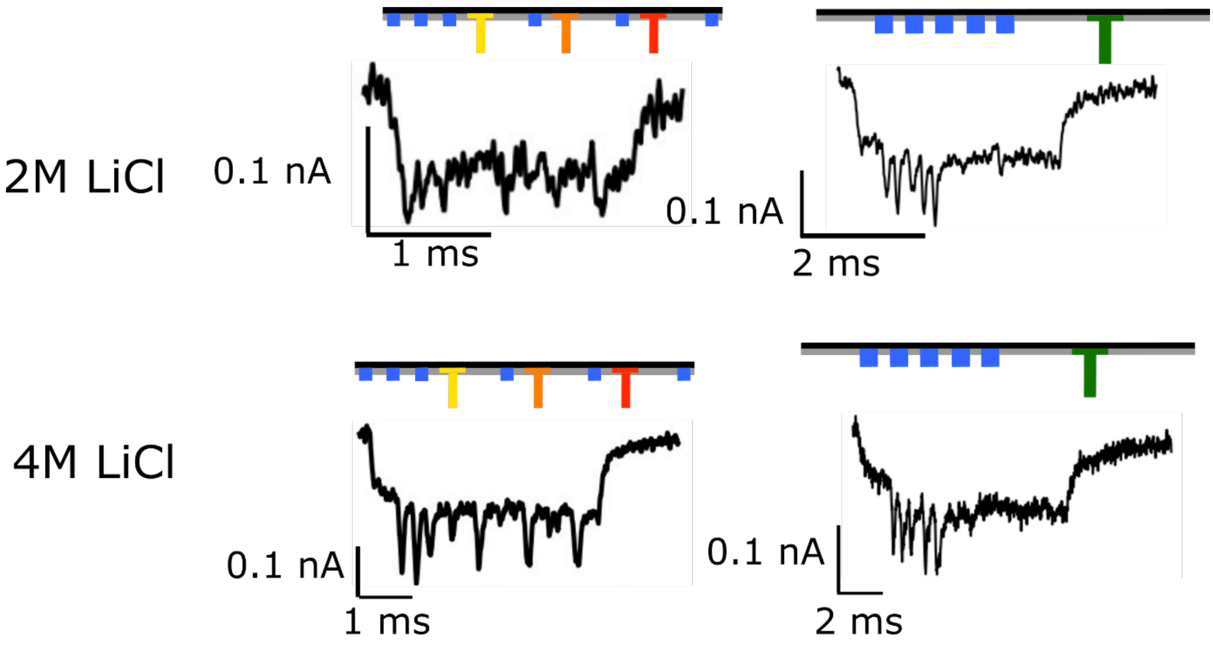

Supplementary Fig. 4 I DNA nanostructures from Figs. 1 and 3 in different salt conditions.
The DNA nanostructure with three overhangs (Fig. 3), contains more dumbbells and more DNA in the overhangs, thus has faster translocations due to a greater overall charge of the molecule. The higher salt concentration acts to slow down the DNA translocation. Thus, this structure is difficult to resolve in 2M but clearly resolved in 4M. For the DNA nanostructure with one overhang and five dumbbells (Figs. 1 and 2) the 2M salt concentration is enough to resolve the structure.

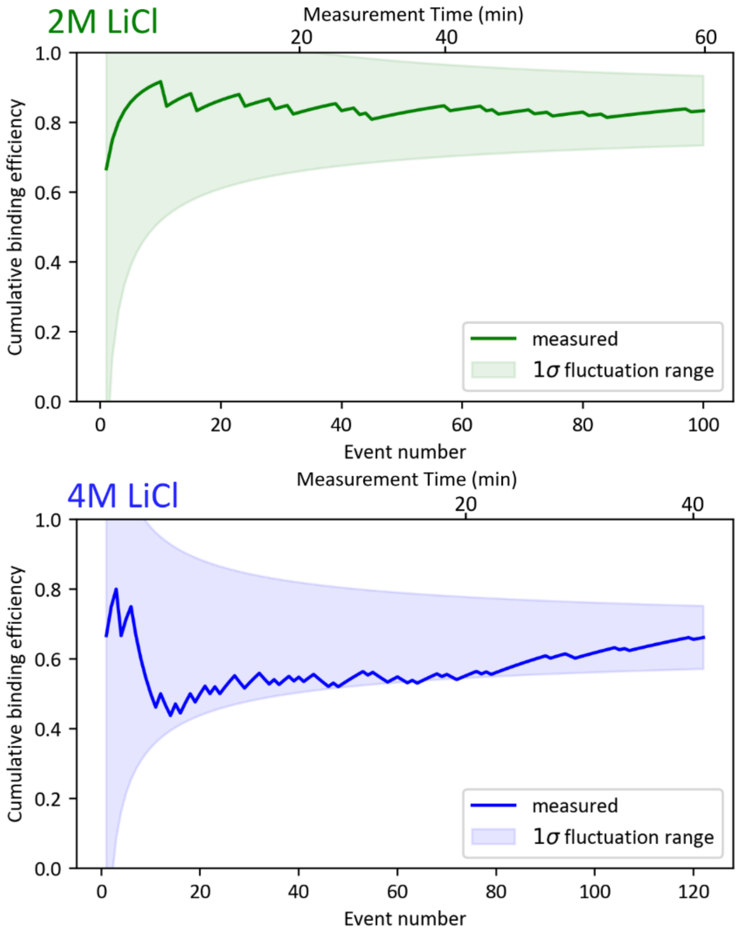

Supplementary Fig. 5 I The DNA nanostructure from Fig. 1a was tested with the purple probe (Figs. 2a and 3c), also called Probe 1 (Fig. 4a) in both 2M and 4M LiCl. The bound dCas9 is found to remain bound for longer at higher efficiencies in 2M than 4M LiCl. This agrees with results previously shown in the literature.

Enzymes are sensitive to salt conditions so the initial binding of the dCas9 probe to the target DNA is performed in low salt conditions similar to physiological conditions. The binding efficiency presented in this paper is the binding efficiency measured in 2M LiCl (as shown in the figure) to remain relatively constant over time. This can lead us to believe the 2M LiCl buffer is not strongly affecting the binding. However, in 4M LiCl the binding is found to decrease over time. It can be seen in the figure that, at the beginning of the measurement in 4M LiCl, the observed binding efficiency is similar to that measured in 2M LiCl, which also suggests that this reflects the real binding efficiency under normal buffer conditions. This also agrees with previous discussions in the literature[2].

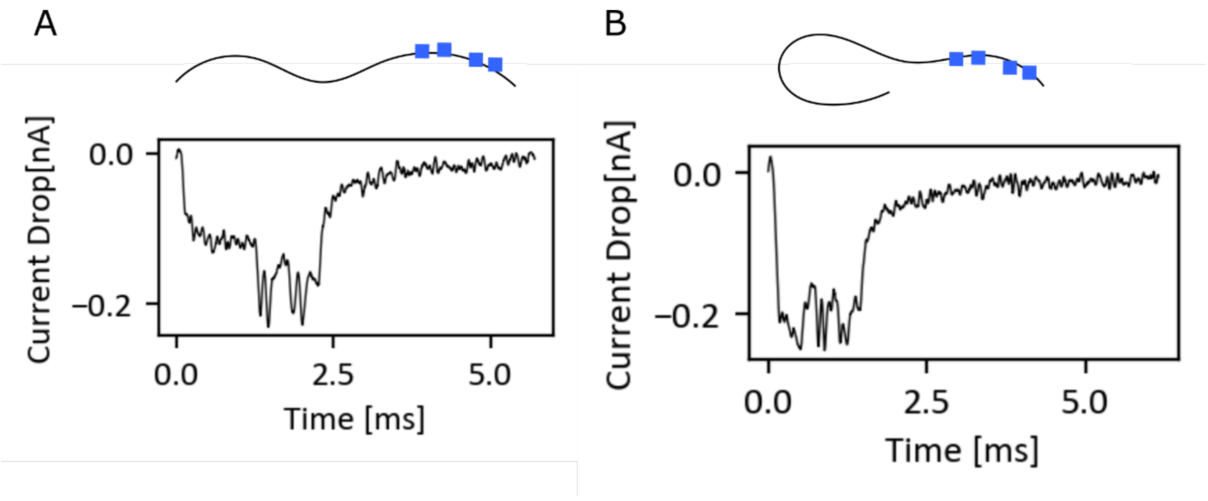

Supplementary Fig. 6 | Unfolded 11011 barcode (a) and folded 11011 barcode (b) nanopore traces taken from the control measurement in Fig. 2b. Because DNA is non-rigid, it can enter the nanopore folded. One can see that when a DNA event is folded, as it is on the right in panel **b**, the time scale becomes shorter compared to that of an unfolded event (panel **a**). One can also see that the initial current drop becomes around double that of the expected DNA baseline of the dsDNA translocating. DNA knotting and folding are commonly observed on solid-state nanopore-sensing systems[3].

References
1. Bell, N. A. & Keyser, U. F. Digitally encoded DNA nanostructures for multiplexed, single-molecule protein sensing with nanopores. *Nature nanotechnology* **11**, 645 (2016).
2. Weckman, N. E. *et al.* Multiplexed DNA Identification Using Site Specific dCas9 Barcodes and Nanopore Sensing. *ACS Sensors* **4**, 2065-2072, doi:10.1021/acssensors.9b00686 (2019).
3. Plesa, C. *et al.* Direct observation of DNA knots using a solid-state nanopore. *Nature Nanotechnology* **11**, 1093-1097, doi:10.1038/nnano.2016.153 (2016).
